# Potash Iodide in Scirrhous Cord

**Published:** 1897-12

**Authors:** M. J. Treacy

**Affiliations:** Veterinarian, U. S. A., Fort Meade, South Dakota


### POTASH IODIDE IN SCIRRHOUS CORD.

By M. J. Treacy, Veterinarian, U. S. A.,
FORT MEADE, SOUTH DAKOTA.

In April last there was presented to me a livery horse, frightfully emaciated, with an unilateral champignon so extensive as to seriously interfere with the propulsion of his posterior limbs. After draining off the effusion, its base measured seventeen inches by eight. There was no discharge, nor could the presence of pus be detected by manipulation or the free use of the exploring trocar and canula.

Having lost two cases from hemorrhage after operation for champignon in the last two years, and thinking naturally that this one presented a very severe test for the kali-iodide treatment, I readily made up my mind to adopt it. In order to tone up his system, his teeth were operated upon, and he received the following, morning and noon: 2 per cent. acid sol. arsenic, ℥x ; tinct. ferri chlor., ℥iij ; strychnia hydrochlor, grs. xxvj ; syrup simp., ℥j. Mix. Sig. Shake; half ounce by syringe, morning and noon, with one drachm of potash iodide every evening. Potash iodide pulv., one drachm in one ounce of water each night. Apply hose to scrotum for one hour, night and morning ; gently exercise two hours daily.

For ten days there was no visible decrease in size of enlargement; after which it rapidly faded away, so to speak ; the horse now went to his usual work, of a very severe kind, and in twenty-six days from commencement of treatment there was no trace of enlargement, the cord not being any thicker than a finger, the horse looking in fine shape. Anticipating a recurrence, I suggested the removal of cord and its attachment to scrotum, to which the owner objected, and up to date, though subjected to the roughest livery work by long drives, irregular feeding and watering, tied up to fences, etc., in cold and rain, often when heated and sweating, as livery horses are habitually used in this country, this horse retains his health so far as scirrhous cord is concerned.

I consider this case an extreme victory for the iodide treatment, but I am inclined to believe that double doses of this drug, from the initial treatment, would have obtained speedier results, as it was apparent that no benefit accrued until the system was at least partially saturated with it. I would try two or three drachms daily until the absorption at least commences.

